# Biomass Allocation in Response to Nitrogen and Phosphorus Availability: Insight From Experimental Manipulations of *Arabidopsis thaliana*

**DOI:** 10.3389/fpls.2019.00598

**Published:** 2019-05-14

**Authors:** Zhengbing Yan, Anwar Eziz, Di Tian, Xiuping Li, Xinghui Hou, Huiyuan Peng, Wenxuan Han, Yalong Guo, Jingyun Fang

**Affiliations:** ^1^Department of Ecology, College of Urban and Environmental Sciences, Peking University, Beijing, China; ^2^College of Life Sciences, Capital Normal University, Beijing, China; ^3^State Key Laboratory of Systematic and Evolutionary Botany, Institute of Botany, Chinese Academy of Sciences, Beijing, China; ^4^Key Laboratory of Plant-Soil Interactions of the Ministry of Education, College of Resources and Environmental Sciences, China Agricultural University, Beijing, China

**Keywords:** allometry, *Arabidopsis thaliana*, biomass allocation, fertilization experiment, N and P availability

## Abstract

Allocation of biomass to different organs is a fundamental aspect of plant responses and adaptations to changing environmental conditions, but how it responds to nitrogen (N) and phosphorus (P) availability remains poorly addressed. Here we conducted greenhouse fertilization experiments using *Arabidopsis thaliana*, with five levels of N and P additions and eight repeat experiments, to ascertain the effects of N and P availability on biomass allocation patterns. N addition increased leaf and stem allocation, but decreased root and fruit allocation. P addition increased stem and fruit allocation, but decreased root and leaf allocation. Pooled data of the five levels of N addition relative to P addition resulted in lower scaling exponents of stem mass against leaf mass (0.983 vs. 1.226; *p* = 0.000), fruit mass against vegetative mass (0.875 vs. 1.028; *p* = 0.000), and shoot mass against root mass (1.069 vs. 1.324; *p* = 0.001). This suggested that N addition relative to P addition induced slower increase in stem mass with increasing leaf mass, slower increase in reproductive mass with increasing vegetative mass, and slower increase in shoot mass with increasing root mass. Further, the levels of N or P addition did not significantly affect the allometric relationships of stem mass vs. leaf mass, and fruit mass vs. vegetative mass. In contrast, increasing levels of N addition increased the scaling exponent of shoot to root mass, whereas increasing levels of P addition exerted the opposite influence on the scaling exponent. This result suggests that increasing levels of N addition promote allocation to shoot mass, whereas the increasing levels of P addition promote allocation to root mass. Our findings highlight that biomass allocation of *A. thaliana* exhibits a contrasting response to N and P availability, which has profound implications for forecasting the biomass allocation strategies in plants to human-induced nutrient enrichment.

## Introduction

Allocation of biomass to different organs is a core component of plant life history and plays a pivotal role in the trade-off between resource acquisition and utilization (Bazzaz and Grace, 1997; Weiner, 2004; Eziz et al., 2017). Nutrient availability is one important factor driving the variability of biomass allocation (Müller et al., 2000; Poorter and Nagel, 2000; Fujita et al., 2014). Quantitative assessments of the impacts of nutrient availability on biomass allocation patterns could be instrumental in elucidating the responses and adaptations of plant growth and ecophysiological processes under global nutrient change (Müller et al., 2000; Shipley and Meziane, 2002; McCarthy and Enquist, 2007).

In recent decades, nitrogen (N) and phosphorus (P) fertilizers and depositions induced by human activities have enhanced the availability of N and P in terrestrial ecosystems (Galloway et al., 2008; Du et al., 2016). As two functionally coupled macroelements in biological systems, N and P have a critical bearing on plant growth and metabolic activities (Lambers et al., 2008; Ågren, 2008), and frequently limit primary productivity across various ecosystems (Aerts and Chapin, 2000; Elser et al., 2007). Previous studies have reported the effects of nutrient or N availability on biomass allocation (e.g., Bazzaz and Grace, 1997; Müller et al., 2000; Shipley and Meziane, 2002; Poorter et al., 2012; Fujita et al., 2014), with little knowledge about the responses of biomass allocation to varying P availability (but see Luo et al., 2016). Thus, considering the tightly coupled relationship between N and P and the imbalance of N vs. P inputs induced by human activities, it is imperative to disentangle the effects of N and P availability on biomass allocation concurrently.

Biomass allocation is often assessed as the fraction of each organ’s biomass to total plant biomass, which potentially reflects plant strategies to cope with resource limitation or disturbance (Poorter and Nagel, 2000; Weiner, 2004; Poorter et al., 2012). For instance, plants grown in nutrient-rich conditions relative to nutrient-poor conditions usually show lower root mass fraction (RMF, the proportion of biomass invested in roots) but higher leaf mass fraction (LMF, the proportion of biomass invested in leaves) and stem mass fraction (SMF, the proportion of biomass invested in stems), thereby increasing light interception and photosynthesis (Müller et al., 2000; de Groot et al., 2002; Poorter et al., 2012). Nutrient deficiency could increase plant reproductive allocation as plants adapted to poor conditions should maintain enough fecundity for survival (Sultan, 2001; Wang et al., 2016). On the other hand, allometric relationships of biomass among different organs are often used to explore the mechanisms of change in biomass allocation (Enquist and Niklas, 2002; Weiner, 2004; Niu et al., 2009; Poorter et al., 2015). Allometric analysis is usually considered in terms of allometric partitioning theory, which states that the responses of proportional biomass allocation to environmental factors are best explained by the changes in plant size along a fixed allometric trajectory (i.e., fixed scaling relationship) (Müller et al., 2000; Weiner, 2004; McCarthy and Enquist, 2007). Through synthesizing data of biomass from global seed plants, Enquist and Niklas (2002) concluded that leaf biomass should scale with the 3/4 power of both stem and root biomass, whereas root biomass should scale isometrically with stem biomass. Weiner et al. (2009) reviewed the relationship between reproductive and vegetative biomass, and revealed that short-lived herbaceous plants often exhibited a simple and linear relationship, whereas larger and longer-lived plants generally had an allometric relationship with a scaling exponent less than one.

To date, whether and how nutrient availability affects biomass allometric relationships among different organs remains contentious. Some studies suggested that biomass allocation patterns could be best explained by the fixed allometric strategies under changing nutrient conditions (Müller et al., 2000; Fortunel et al., 2009; Peng and Yang, 2016). For instance, Müller et al. (2000) observed that the allometric scaling relationship of aboveground against belowground biomass was unaffected by N addition in 21 out of 27 species. Similarly, through field investigations or meta-analysis, several studies have found that allometric relationships between aboveground and belowground biomass were reported to be isometric and rarely altered by nutrient availability (Fortunel et al., 2009; Yang et al., 2009; Peng and Yang, 2016). However, some studies emphasized that the bivariate scaling exponent of biomass between organs varied with nutrient availability (Shipley and Meziane, 2002; Huang et al., 2009; Luo et al., 2016; Yan B.G. et al., 2016). For instance, Shipley and Meziane (2002) found that the scaling exponent of aboveground against belowground biomass for 22 herbaceous species was altered by N addition. In an arid-hot grassland, the allometric relationship between stem and leaf biomass varied across a soil nutrient gradient, which influenced plant adaptation and distribution (Yan B.G. et al., 2016). These conflicting findings warrant more in-depth studies before drawing general conclusions. Besides, effects of N and P availability on the allometric relationships of biomass among different organs are rarely compared in concert (but see Luo et al., 2016).

In this study, we examined the effects of N and P availability on the biomass allocation of *Arabidopsis thaliana*, which is a model annual plant for molecular biological studies (Meinke et al., 1998). We conducted a series of greenhouse fertilization experiments with five levels of N and P additions and eight repeat experiments. Here we chose *A. thaliana* as the target species, mainly because it has a short lifespan and could be planted for several consecutive experiments during a short period. Specifically, this study aimed to address the following questions: (i) how do N and P availability affect the biomass allocation fractions? and (ii) how do N and P availability regulate the allometric relationships of biomass among different organs? Considering the differential roles of N and P in physiological functions and metabolic processes in plants, we hypothesize that (i) N addition increase leaf and stem allocation but decrease root and fruit allocation, whereas P addition increase stem and fruit allocation but decrease root and leaf allocation; (ii) N addition relative to P addition results in lower scaling exponents of stem mass against leaf mass, fruit mass against vegetative mass, and shoot mass against root mass.

## Materials and Methods

### Growth Conditions and Experimental Design

In this study, we performed the greenhouse N and P fertilization experiments using *A. thaliana* from ecotype “Columbia” (Yan et al., 2015; Yan Z.B. et al., 2016; Yan et al., 2018), and repeated eight such experiments consecutively. We carried out all these experiments under nearly the same greenhouse conditions and similar experimental processes. These experiments were implemented in a phytotron with 16-h light/8-h dark photoperiod at approximately 20°C and 60–70% relative humidity. Light was provided by one yellow sodium fluorescent lamp and five white fluorescent lamps. Before sowing, seeds were surface sterilized using 70% (v/v) ethanol-0.5% (v/v) Tween 20 and then stratified in a 0.1% (w/v) agar solution at 4°C in the dark for 4 days. Hereafter, seeds were sown in plastic pots with 24 small divisions in favor of raising 24 individuals per pot. These pots were filled with sterilized vermiculite (medium particle size) containing few mineral nutrients, and the base of each pot was periodically watered by respective nutrient solutions with various N and P concentrations. Altogether, there were 27 pots from nine nutrient treatments with three replicates for each. To diminish the impacts of microenvironment, we randomly rearranged these pots every 2 days.

The experiments included nine nutrient treatments, which consisted of five levels of N addition (1, 2, 4, 8, and 12 mmol N L^-1^, added as NH_4_NO_3_) at the intermediate level of P addition (0.25 mmol P L^-1^) and five levels of P addition (0.0625, 0.125, 0.25, 0.5, and 1.0 mmol L^-1^, added as KH_2_PO_4_ and NaH_2_PO_4_) at the intermediate level of N addition (4 mmol N L^-1^), respectively. We designed the levels of N and P addition mainly based on the dose-responses in our preliminary experiments (Yan et al., 2015). The intermediate level of nutrient addition was defined as the level when the shoot mass reached to the maximum value along the increasing levels of nutrient addition. The five levels of N addition were indicated by N1, N2, N3, N4, and N5, and the five levels of P addition were indicated by P1, P2, P3, P4, and P5. The intermediate levels of N and P additions were indicated by N3 and P3, respectively. N addition experiments shared the same treatment of N3P3 with P addition experiments. All nutrient solutions contained the same concentrations of other macro- and microelements (i.e., all nutrient solutions per litter equally contained 2 mmol CaCl_2_, 0.75 mmol K_2_SO_4_, 0.65 mmol MgSO_4_, 0.1 mmol Fe-EDTA, 0.01 mmol H_3_BO_3_, 1 μmol MnSO_4_, 1 μmol ZnSO_4_, 0.1 μmol CuSO_4,_ and 0.035 μmol Na_2_MoO_4_) except for N and P. The pH of each nutrient solution was adjusted to 5.8. Elemental composition and concentrations of the nutrient solutions conformed to Hoagland’s formula (Hoagland and Arnon, 1950), and were then modified according to our preliminary experiments (Yan et al., 2015).

### Sampling and Measurement

We sampled plants at the end of main stem inflorescences in *A. thaliana* when the fruits are mostly mature (designated as the “fruit maturity stage”). Sampling date was adjusted to match the individual ontogenetic stage, considering that plant growth rates varied among treatments. Leaves, stems and fruits were divided, and then oven-dried at 65°C to constant weight. Aboveground mass of each individual plant was obtained by pooling the mass of leaves, stems, and fruits together. Vegetative mass of each individual plant was obtained by pooling the mass of leaf and stem together. Biomass allocation fraction for each organ was calculated by the organ’s mass as a fraction of aboveground mass. Four individuals with almost similar growth for each replicate were sampled and measured. Root biomass allocation was not analyzed in current fertilization experiments, because of lack of root biomass data. To replenish the understanding of root biomass allocation under N and P additions, we used biomass data including roots from our preliminary experiment to examine effects of N and P additions on root to shoot (R:S) ratio and allometric relationship of shoot mass against root mass in *A. thaliana*.

### Data Analyses

We conducted two types of analyses in this study. Firstly, changes of biomass allocation fractions along the levels of N and P additions were examined using one-way analysis of variance (ANOVA) and the least significant difference *post hoc* test. Secondly, we performed standardized major axis (SMA) regression analyses to explore allometric relationships of biomass among different organs under influences of the type and level of N and P additions. A likelihood ratio test was used to indicate the heterogeneity of the scaling exponents among different groups (Warton et al., 2006). Effects of the type of nutrient addition on those relationships were analyzed using pooled data of the five levels of N or P addition. Effects of the levels of nutrient addition on those relationships were explored using data from each level of N or P addition. Data was log_10_-transformed before SMA regression analysis. We conducted all statistical analyses using combined data from the eight repeat experiments in R 2.15.2 (R Development Core Team, 2012).

## Results

**FIGURE 1:**
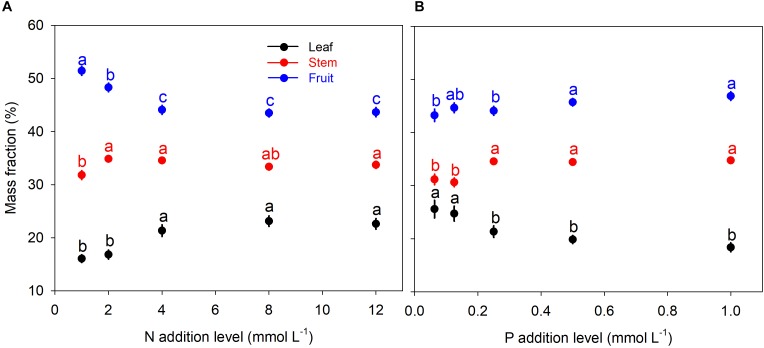
Biomass allocation fractions under different levels of N and P additions. **(A)** N additions; **(B)** P additions. Points and error bars denote the means and standard errors of biomass allocation fractions, respectively. Different letters above the error bars indicate significant difference (*p* < 0.05) among five levels of N or P addition based on one-way analysis of variance (ANOVA) and the least significant difference *post hoc* test.

**FIGURE 2:**
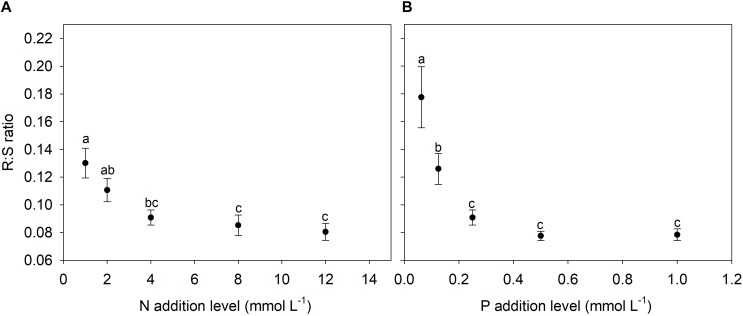
Changes in root to shoot (R:S) ratio with levels of N and P additions. **(A)** N additions; **(B)** P additions. Points and error bars denote the means and standard errors of R:S ratio, respectively. Different letters above the error bars indicate significant difference (*p* < 0.05) among five levels of N or P addition based on one-way analysis of variance (ANOVA) and the least significant difference *post hoc* test.

N and P availability exhibited contrasting effects on biomass allocation fractions of *A. thaliana* (Figure 1). With increasing levels of N addition, LMF, and SMF initially increased and then leveled off at higher addition levels, whereas root to shoot (R:S) ratio and fruit mass fraction (FMF) initially decreased sharply and then leveled off at higher levels of N addition (Figure 1A, 2A and Supplementary Table S1). In contrast, with increasing levels of P addition, LMF and R:S ratio showed a rapid decrease at lower addition levels and then leveled off, whereas SMF and FMF presented a rapid increase at lower addition levels and then leveled off (Figure 1B, 2B and Supplementary Table S1).

**FIGURE 3:**
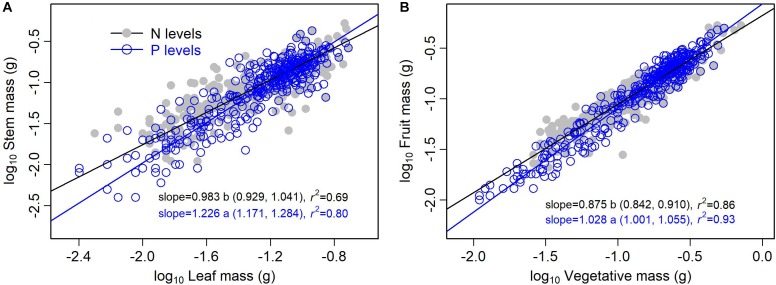
Effects of the type of nutrient addition (i.e., N additions vs. P additions) on the allometric relationships for **(A)** stem mass vs. leaf mass, and **(B)** fruit mass vs. vegetative mass. SMA regression is used to determine the significant line (*p* < 0.05). Relationships for N (or P) levels are examined using data pooled from the five levels of N (or P) addition. Numbers in square brackets are the lower and upper 95% confident intervals of the SMA slopes. Different letters after scaling slopes indicate significant difference (*p* < 0.05) based on a likelihood ratio test.

**FIGURE 4:**
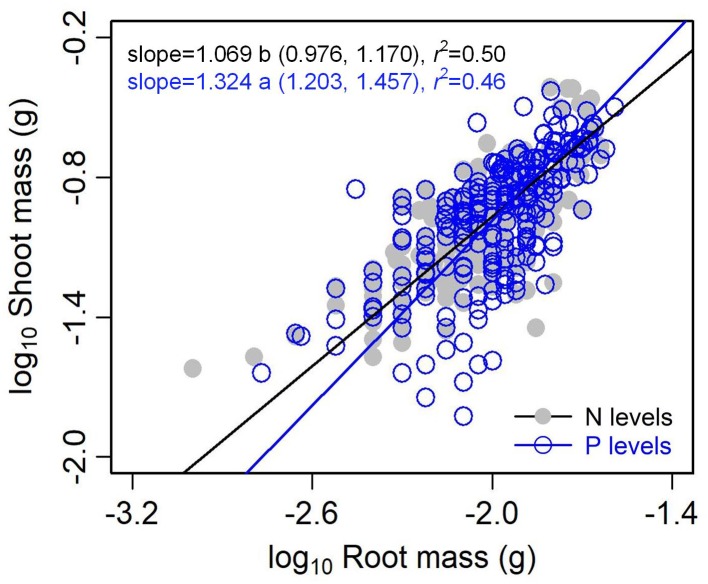
Effects of the type of nutrient addition (i.e., N addition vs. P addition) on the allometric relationships between shoot mass and root mass. SMA regression is used to determine the significant line (*p* < 0.05). Relationships for N (or P) levels are examined using data pooled from the five levels of N (or P) addition. Numbers in square brackets are the lower and upper 95% confident intervals of the RMA slopes. Different letters after scaling slopes indicate significant difference (*p* < 0.05) based on a likelihood ratio test.

The type of nutrient addition regulated allometric relationships of biomass among different organs (Figure 3, 4). In comparison with pooled data of the five levels of P addition, pooled data of the five levels of N addition showed significantly lower scaling exponents of stem mass against leaf mass (0.983 vs. 1.226; *p* = 0.000), fruit mass against vegetative mass (0.875 vs. 1.028; *p* = 0.000), and shoot mass against root mass (1.069 vs. 1.324; *p* = 0.001) (Figure 3, 4). These results suggested that N additions relative to P additions caused slower increase in stem mass with increasing leaf mass, slower increase in reproductive mass with increasing vegetative mass, and slower increase in shoot mass with increasing root mass.

**FIGURE 5:**
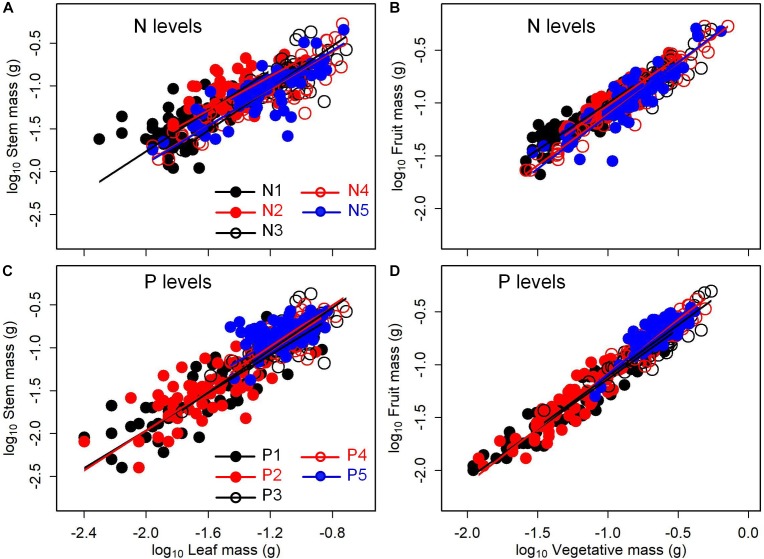
Effects of the levels of N and P addition on the allometric relationships for **(A,C)** stem mass vs. leaf mass, and **(B,D)** fruit mass vs. vegetative mass. SMA regression is used to determine significant line (*p* < 0.05) for each level of N or P addition.

The levels of N or P addition exerted little influences on allometric relationships of stem mass vs. leaf mass and fruit mass vs. vegetative mass (Table 1 and Figure 5). Scaling exponents and normalization constants of stem mass against leaf mass showed the insignificant difference among the five levels of N (or P) addition (Table 1 and Figure 5A,C). Similarly, scaling exponents and normalization constants of fruit mass against vegetative mass also exhibited the insignificant difference among the five levels of N (or P) addition (Table 1 and Figure 5B,D). In contrast, increasing levels of N addition increased the scaling exponent of shoot to root mass, whereas increasing levels of P addition exerted the opposite influence on the scaling exponent (Table 2). This result suggested that increasing levels of N addition promoted allocation to shoot mass, whereas increasing levels of P addition promoted allocation to root mass.

**Table 1:** Summary of the SMA regressions about the allometric relationships of biomass among different organs [e.g., log_10_ Stem mass = *α*^∗^log_10_ (Leaf mass)+*β*] for various levels of N or P addition.

	Stem mass vs. Leaf mass			Fruit mass vs. Vegetative mass		
	*α*_SMA_ (95% CI)	*β*_SMA_ (95% CI)	*r*^2^	*α*_SMA_ (95% CI)	*β*_SMA_ (95% CI)	*r*^2^
**N addition (mmol L^–1^)**		
1	1.178a(0.983, 1.411)	0.593a(0.223, 0.962)	0.36	0.887a(0.794,0.990)	–0.113a (–0.236, 0.010)	0.76
2	0.994a(0.824, 1.200)	0.308a(0.044, 0.573)	0.29	0.922a(0.827,1.028)	–0.101a (–0.193, –0.009)	0.77
4	1.235a(1.063, 1.434)	0.455a(0.247, 0.664)	0.56	0.972a(0.876,1.078)	–0.134a (–0.207, –0.061)	0.79
8	1.078a(0.967, 1.200)	0.263a(0.113, 0.412)	0.81	0.969a(0.903,1.039)	–0.135a (–0.197, –0.074)	0.92
12	1.101a(0.945, 1.282)	0.298a(0.081, 0.515)	0.62	1.009a(0.901,1.130)	–0.109a (–0.211, –0.007)	0.79
**P addition (mmol L^–1^)**		
0.0625	1.102a(0.943, 1.288)	0.240a(–0.050,0.530)	0.70	0.947a (0.866, 1.035)	–0.188a (–0.301, –0.075)	0.90
0.125	1.165a(1.017, 1.335)	0.366a (0.113, 0.620)	0.66	1.042a (0.962, 1.129)	–0.041a (–0.144, 0.062)	0.88
0.25	1.235a(1.063, 1.434)	0.455a (0.247, 0.664)	0.56	0.972a (0.876, 1.078)	–0.134a (–0.207, –0.061)	0.79
0.5	1.201a(1.012, 1.424)	0.456a (0.234, 0.679)	0.44	1.013a (0.898, 1.142)	–0.063a (–0.141, 0.015)	0.73
1	1.072a(0.879, 1.308)	0.355a (0.115, 0.595)	0.24	1.080a (0.947, 1.232)	–0.008a (–0.101, 0.086)	0.67


*Lower and upper 95% confidence intervals (CIs) of the SMA slopes are presented for all relationships with *p* < 0.05. The same letter denotes the insignificant difference (*p* > 0.05) of the scaling exponent and normalization constant among the five levels of N or P addition based on a likelihood-ratio test. Data are log_*10*_-transformed before analysis.*

**Table 2:** Summary of the SMA regressions about the allometric relationships between shoot mass and root mass [e.g., log_10_ Shoot mass = α^∗^log_10_ (Root mass)+β] for various levels of N or P addition.

	*α*_SMA_ (95% CI)	*β*_SMA_ (95% CI)	*r*^2^
**N addition (mmol L^-1^)**		
1	0.864 bc (0.687, 1.086)	0.633 bc (0.213, 1.054)	0.35
2	0.758 c (0.632, 0.910)	0.500 c (0.220, 0.781)	0.60
4	0.806 bc (0.656, 0.990)	0.672 bc (0.335, 1.010)	0.53
8	1.340 a (1.125, 1.600)	1.778 a (1.302, 2.254)	0.66
12	1.124 ab (0.935, 1.351)	1.358 ab (0.926, 1.790)	0.66
**P addition (mmol L^-1^)**		
0.0625	1.812 a (1.398, 2.347)	2.408 a (1.451, 3.365)	0.40
0.125	1.210 ab (0.993, 1.473)	1.366 ab (0.874, 1.858)	0.58
0.25	0.806 c (0.656, 0.990)	0.672 b (0.335, 1.010)	0.53
0.5	0.781 c (0.640, 0.953)	0.703 b (0.399, 1.006)	0.51
1	1.018 bc (0.890, 1.165)	1.155 b (0.881, 1.429)	0.79


*Lower and upper 95% confidence intervals (CIs) of the SMA slopes are presented for all relationships with *p* < 0.05. The same letter denotes the insignificant difference (*p* > 0.05) of scaling exponent and normalization constant among five levels of N or P addition based on a likelihood-ratio test. Data are log_*10*_-transformed before analysis.*

## Discussion

### N and P Availability Differently Altered Biomass Allocation Fractions

In this study, we found that N addition increased LMF but decreased FMF, whereas P addition showed the opposite effects on these two. Similarly, Wang et al. (2016) also found that N addition induced the decrease in reproductive allocation of *Chloris virgata*. Fujita et al. (2014) found that plants distributed in P-deficient communities showed low investment of resources in sexual reproductive tissues. One potential cause for a contrasting response may be that N and P have differential roles in physiological functions and metabolic processes in plants. Large proportion of acquired N is allocated into photosynthetic apparatus (Yasumura et al., 2007; Ghimire et al., 2017), and N could then more strongly constrain photosynthesis relative to reproduction (Fortunel et al., 2009). Accordingly, N addition could promote carbon assimilation capacity and leaf expansion (Lambers et al., 2008), resulting in an increase in the LMF. In contrast, plant invests up to *c.* 50–60% of acquired P in reproductive tissues, which is larger for P than for N (Güsewell, 2004; Kerkhoff et al., 2006). Accordingly, P deficiency tends to reduce the reproductive allocation (Güsewell, 2004; Fujita et al., 2014).

In addition, differences in the phenological response of plants to N and P availability could regulate biomass allocation pattern in plants (Bazzaz and Grace, 1997; McConnaughay and Coleman, 1999). The delay of flowering implies that plants postpone the switch from vegetative growth to reproductive growth, resulting in the decreasing allocation of biomass to reproductive organs (Zhang et al., 2014). Our results showed that low P availability delayed the flowering date in *A. thaliana* (Supplementary Figure S2), which may further decrease reproductive allocation. In contrast, we found that N availability exerted insignificant effect on flowering date of *A. thaliana* (Supplementary Figure S2). Several other studies found that the responses of plant flowering date and reproductive allocation fractions to N addition differed among various species (Tian et al., 2012; Zhang et al., 2014). Thus, observed pattern regarding the effects of N availability on reproductive allocation fraction of *A. thaliana* may be species-specific and should be cautioned when extending to other species.

Consistent with previous studies regarding the effects of nutrient availability on SMF (de Groot et al., 2002; Poorter et al., 2012), we found that both N and P additions increased SMF of *A. thaliana*. Generally, stem is regarded as an effectively luxury organ for plants grown in nutrient poor environments, and thus plants distributed in such conditions usually reduce the SMF (Yan B.G. et al., 2016). In contrast, plants grown in high nutrient supply often need to allocate more biomass into stem in order to provide mechanical supports for the enhanced aboveground biomass. Moreover, nutrient addition that induced crowded conditions in field plant populations could promote SMF for coping with light competition (Niu et al., 2008; Zhang et al., 2014; Yan B.G. et al., 2016). R:S ratio of *A. thaliana* decreased with increasing levels of N and P additions, which was in accordance with previous findings (Bazzaz and Grace, 1997; Müller et al., 2000; de Groot et al., 2002; Poorter et al., 2012).

Besides, we found another intriguing result that pattern of biomass allocation did not change after moderate additions of N and P (Figure 1). Effects of N and P additions on biomass allocation fractions were only significant when N and P availability limited the plant growth (Figure 1). Beyond the threshold of nutrient availability, further addition of N or P exited insignificant influence on biomass allocation fractions (Figure 1). Thus, effect of nutrient addition on biomass allocation pattern should depend on substrate nutrient limitation status.

Peng et al. (2019) has conducted a similar N and P fertilization experiment to explore the responses of stoichiometry and nutrient resorption efficiency of *Amaranthus mangostanus*. Using their biomass data, we further analyzed effects of N and P additions on biomass allocation of *A. mangostanus*. Results showed that N and P additions decreased R:S ratio, whereas N (or P) addition increased (or decreased) LMF (Supplementary Figures S1a,b). These results are in accordance with our study. However, response of SMF to N and P additions was very complex, and was adjusted by the interaction of N and P additions (Supplementary Figure S1c). In contrast with N addition, P addition exerted larger and positive influence on FMF (Supplementary Figure S1d), which is partly consistent with our findings. Overall, effects of N and P additions on biomass allocation to root, leaf and reproductive tissues of *A. thaliana* is mostly applicable to those of *A. mangostanus*. Further study is needed to test the generality of biomass allocation pattern across species with different life strategies.

### N and P Availability Contrastingly Influenced Biomass Allometric Relationships

Shift in scaling exponents of biomass allometric relationships suggests an independent change in functional tradeoff among organs, which is very meaningful to understand how plant balance fixed biomass in limitation of different available resources (Weiner, 2004; Poorter et al., 2015). In this study, we found that the type of nutrient addition regulated scaling exponent of stem mass against leaf mass. That is, allometric relationship of stem mass against leaf mass was nearly isometric for pooled data of the five levels of N addition, whereas the relationship was allometric with scaling exponent of 1.226 (i.e., stem ∝ leaf^1.226^) for pooled data of the five levels of P addition. This difference in the scaling exponent might be attributed to the disproportionate increase in leaf mass under N addition relative to P addition. Both LMF and SMF increased with increasing level of N addition, while LMF decreased and SMF increased with increasing level of P addition. Thereby, plants under P additions relative to N additions should exhibit a lower investment in leaf mass per additional unit of stem mass accumulated. These findings indicated that there was plasticity in allometric relationship between stem mass and leaf mass for *A. thaliana*. In addition, previous important recognition of isometry between leaf and stem biomass for herbaceous species (Niklas and Cobb, 2006; Enquist et al., 2007) didn’t conform to the result under P additions. As *A. thaliana* is a rosette forb and primary function of its stem is to support reproductive tissues, allometric relationship between stem mass and leaf mass might thus be different from previous traditional viewpoint.

Relationship between reproductive and vegetative mass is a fundamental component of plants’ reproductive strategy (Reekie and Bazzaz, 2005). Multiple environmental factors might change allometric coefficients of the relationship (Weiner et al., 2009). In our study, we found that the type of nutrient addition significantly regulated scaling exponent of the relationship. Namely, scaling exponent of fruit mass (i.e., reproductive mass) against vegetative mass for pooled data of the five levels of N addition was significantly lower than that for pooled data of the five levels of P addition (0.875 vs. 1.028, *p* < 0.05). This difference might be attributed to a contrasting roles of N and P on the trade-off between reproductive and vegetative growth. N addition facilitated a higher investment of biomass in vegetative organ, whereas P addition favored a higher investment of biomass in reproductive organ (Lambers et al., 2008; Fujita et al., 2014; Kitayama et al., 2015). Thereby, plants under N addition relative to P addition should exhibit a lower investment in reproductive mass per additional unit of vegetative mass accumulated. These findings further indicated that there was plasticity in the allometric relationship between reproductive mass and vegetative mass for *A. thaliana*.

Compared with the type of nutrient addition, the levels of N or P addition exerted little impacts on scaling exponents and normalization constants of allometric relationships of stem mass vs. leaf mass and fruit mass vs. vegetative mass (Table 1 and Figure 5). Similarly, through conducting experimental manipulations with species from the Mediterranean old-field succession, Müller et al. (2000) found common allometric relationships of biomass among different plant structures under several levels of N addition. Fortunel et al. (2009) found that allometric relationships between reproductive and leaf biomass had no difference in either slope or normalization constant among different levels of N addition, suggesting the little plasticity of reproductive strategies. Peng and Yang (2016) synthesized data from global N fertilization experiments and found that the levels of N addition didn’t alter allometric relationship between aboveground and belowground biomass. However, we found that increasing levels of N addition increased scaling exponent of shoot mass to root mass, whereas increasing levels of P addition exerted the opposite influence on the scaling exponent (Table 2). Consistently, Luo et al. (2016) found that scaling exponent of shoot to root mass increased with N:P supply ratio. These results suggested that plants under higher N:P supply ratio exhibited a bias toward elevated aboveground allocation, because root growth is more severely suppressed than shoot growth under P limitation (Güsewell, 2005; Venterink and Güsewell, 2010).

## Conclusion

This study ascertained contrasting effects of N and P availability on biomass allocation of *A. thaliana*. N addition increased leaf and stem allocation, but decreased root and fruit allocation. P addition increased stem and fruit allocation, but decreased root and leaf allocation. Pooled data of the five levels of N addition relative to P addition resulted in lower scaling exponents of stem mass against leaf mass, fruit mass against vegetative mass, and shoot mass against root mass. Further, the levels of N or P addition did not significantly affect allometric relationships of stem mass vs. leaf mass, and fruit mass vs. vegetative mass. In contrast, increasing levels of N addition increased scaling exponent of shoot to root mass, whereas increasing levels of P addition exerted opposite influence on scaling exponent. Our findings highlight that biomass allocation of *A. thaliana* is differentially influenced by N and P availability, which could foster the understanding about response and acclimation of biomass allocation strategies in plants to varying nutrient conditions. We also point out that the types and levels of nutrient availability should be considered, respectively, when exploring effects of nutrient availability on biomass allometric relationships. However, whether the observed pattern in *A. thaliana* can be extended to other species with different functional traits necessitates further studies.

## Author Contributions

JF designed the research. ZY and JF performed the research and analyzed the data. All authors wrote the manuscript.

## Conflict of Interest Statement

The authors declare that the research was conducted in the absence of any commercial or financial relationships that could be construed as a potential conflict of interest.
