# Supplementary material for: Biomass Allocation in Response to Nitrogen and Phosphorus Availability: Insight From Experimental Manipulations of Arabidopsis thaliana
Source: Front Plant Sci. 2019 May 14;10:598. doi: 10.3389/fpls.2019.00598 (PMC6528069; doi:10.3389/fpls.2019.00598)

**SUPPORTING INFORMATION**

**Biomass allocation in response to nitrogen and phosphorus availability: insight from experimental manipulations of *Arabidopsis thaliana***

Zhengbing Yan^1^, Anwar Eziz^1^, Di Tian^2^, Xiuping Li^1^, Xinghui Hou^3^, Huiyuan Peng^4^, Wenxuan Han^4^, Yalong Guo^3^, Jingyun Fang^1*^

1 Department of Ecology, College of Urban and Environmental Sciences, Peking University, Beijing 100871, China

2 College of Life Sciences, Capital Normal University, Beijing, 100048, China

3 State Key Laboratory of Systematic and Evolutionary Botany, Institute of Botany, Chinese Academy of Sciences, Beijing 100093, China

4 Key Laboratory of Plant-Soil Interactions of the Ministry of Education, College of Resources and Environmental Sciences, China Agricultural University, Beijing, China

*Corresponding author J.Y. Fang

Email: [jyfang@urban.pku.edu.cn](mailto:jyfang@urban.pku.edu.cn)

Tel: +86-10-6276 5578

FAX: +86-10-6275 6560

**This file includes:**

**Table S1**

**Figures S1-S2**

**Table S1** Summary of the one-way analysis of variance (ANOVA) of biomass allocation fractions under N and P additions. Abbreviations: LMF, leaf mass fraction; SMF, stem mass fraction; FMF, fruit mass fraction; df, degrees of freedom; MS, mean squares.

|  | LMF | SMF | FMF |
| --- | --- | --- | --- |
| *N addition* |  |  |  |
| df | 4 | 4 | 4 |
| MS | 259.32 | 34.68 | 301.39 |
| F value | 12.23 | 3.30 | 18.31 |
| *p* value | <0.001 | 0.013 | <0.001 |
| *P addition* |  |  |  |
| df | 4 | 4 | 4 |
| MS | 223.91 | 97.29 | 46.41 |
| F value | 6.78 | 8.37 | 2.46 |
| *p* value | <0.001 | <0.001 | 0.050 |

**Figure S1** Biomass allocation of *Amaranthus mangostanus* under different levels of N and P additions. Effects (F value) of N and P additions and their interaction (N×P) on biomass allocation were presented in each panel. F and *p* values (****p*<0.001; ***p*<0.01; **p*<0.05) were determined by two-way analysis of variance.


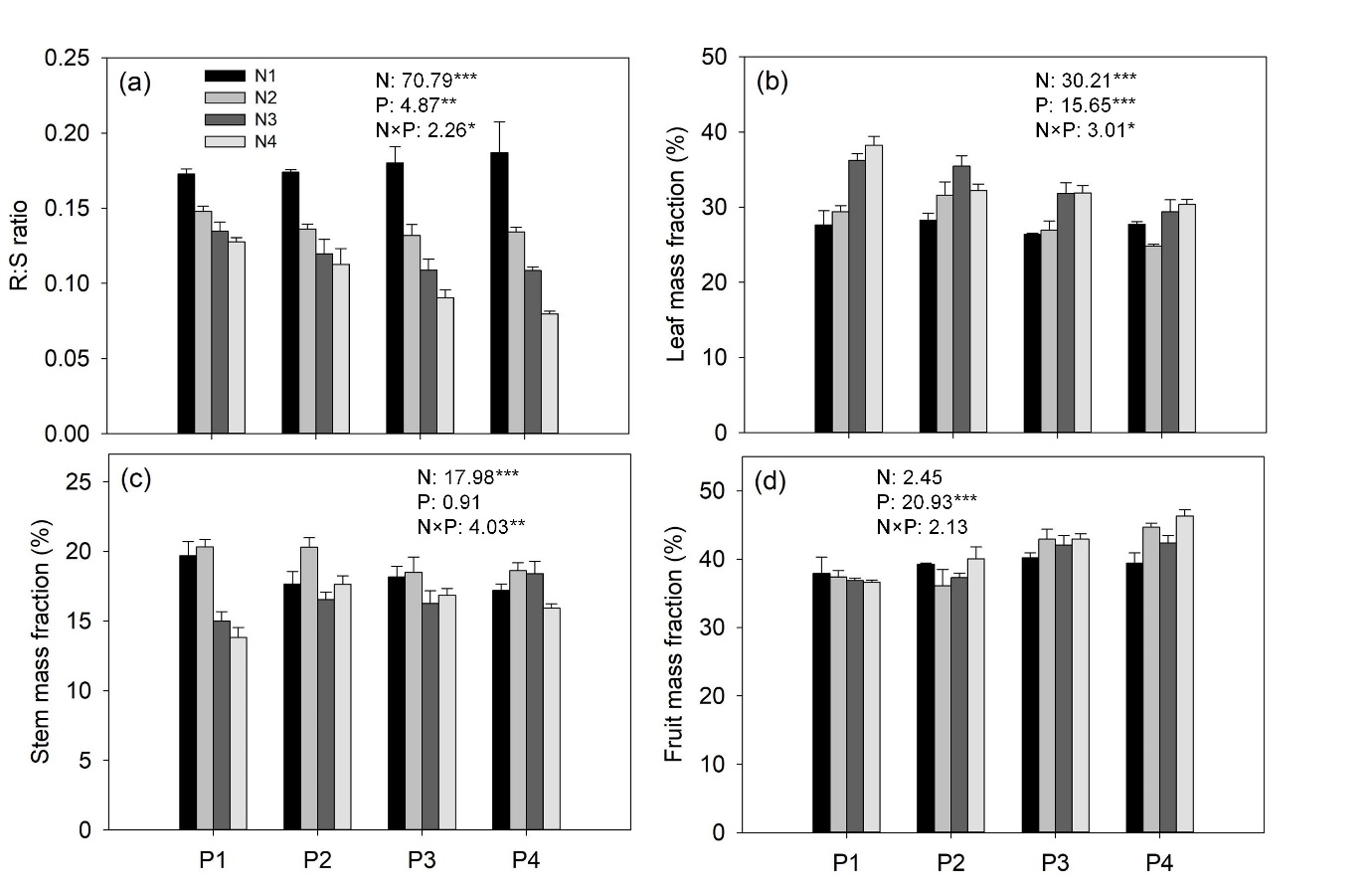


**Figure S2** Changes in the flowering time of *A. thaliana* with the levels of N and P additions. Points and error bars denote the means and standard errors of flowering time, respectively. Different letters above the error bars indicate significant difference (*p*<0.05) among the five levels of N or P addition based on one-way analysis of variance (ANOVA) and the least significant difference post hoc test.


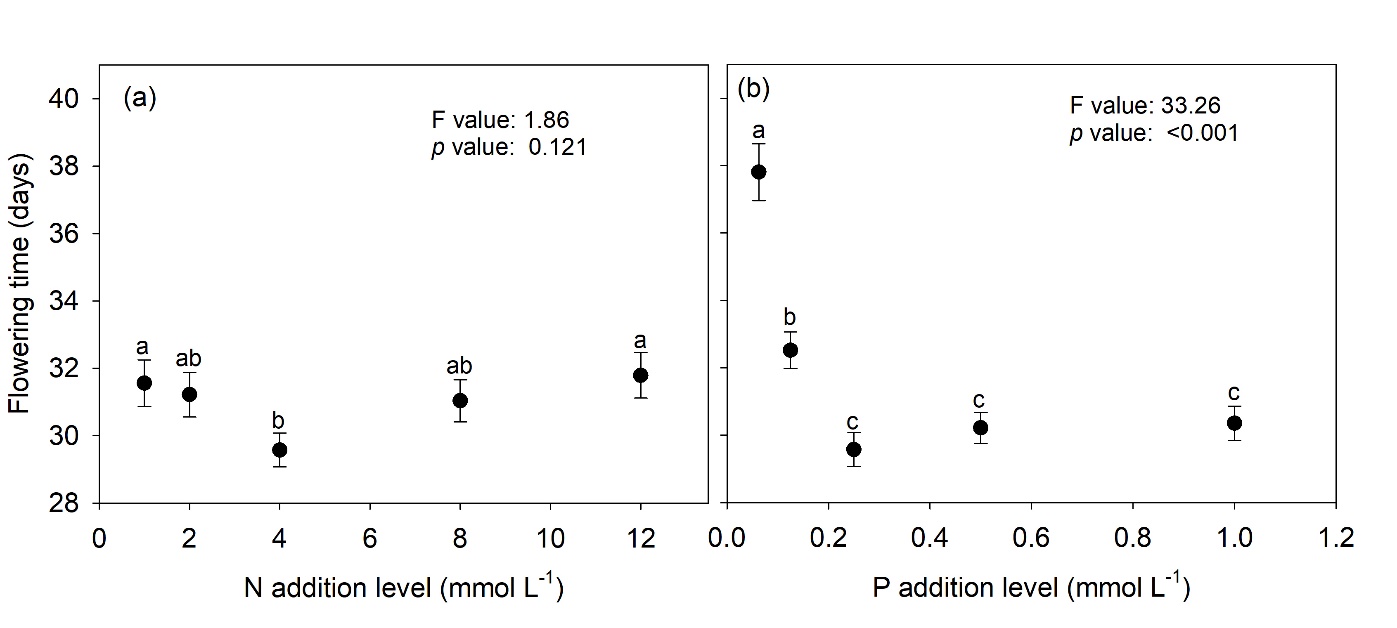

Supplement: Supplementary file 1 [file Data_Sheet_1.docx]
